# Under‐Five Mortality in Humanitarian Emergencies: A Comparison of Face‐to‐Face and Mobile Phone Survey Estimates From Burkina Faso

**DOI:** 10.1111/tmi.70176

**Published:** 2026-06-11

**Authors:** Kassoum Dianou, Bruno Masquelier, Stéphane Helleringer, Bruno Lankoandé, Adramane B. Soura, Oumar Billa, Yacouba Compaoré, Georges Reniers

**Affiliations:** ^1^ Centre for Demographic Research (DEMO) Louvain University Louvain‐La‐Neuve Belgium; ^2^ Département Population et Santé, Institut Supérieur Des Sciences de la Population (ISSP) Joseph Ki‐Zerbo University Ouagadougou Burkina Faso; ^3^ Division of Social Science New York University Abu Dhabi UAE; ^4^ Institute for Global Health Northwestern University Chicago USA; ^5^ Centre National de la recherche Scientifique et Technologique (CNRST)/Institut de recherche en Sciences de la santé (IRSS/Département Biomédical et Santé Publique (BIOMED)) Ouagadougou Burkina Faso; ^6^ Department of Population Health London School of Hygiene and Tropical Medicine London UK

**Keywords:** Burkina Faso, child mortality, data collection methods, humanitarian crisis, LMICs, mobile phone surveys

## Abstract

**Objectives:**

We evaluate the utility of mobile phone surveys (MPS) for estimating child mortality in Burkina Faso, where armed conflict and forced displacements hamper traditional face‐to‐face data collection on an important share of the territory.

**Methods:**

We estimated under‐five mortality from truncated birth histories collected in an MPS from September 2021 to October 2022. The sample consisted of telephone numbers collected in a prior face‐to‐face survey and an additional set of numbers generated through random digit dialling (RDD). We compared regionally disaggregated mortality estimates from the MPS with those from the 2021 Demographic and Health Survey (DHS), which could not cover parts of the national territory due to insecurity.

**Results:**

Of 52,650 attempted phone numbers, 11,387 women of reproductive age were interviewed. Of these, 8077 were reached through RDD (36% lived in conflict‐affected areas at the time of data collection) and 3310 via previously collected numbers (42% lived in conflict‐affected areas). In regions not directly affected by conflict, under‐five mortality rates have remained relatively stable over time: from 45 deaths per 1000 live births in 2018–2019 (95% CI: 36–54) to 58 in 2020–2022 (95% CI: 46–69). This finding mirrors the trend observed in the DHS data. In regions affected by armed conflict, however, the MPS data indicate a substantial increase in under‐five mortality: From 47 deaths per 1000 live births in 2018–2019 (95% CI: 38–57) to 82 in 2020–2022 (95% CI: 67–98). This mortality reversal in conflict‐affected areas is not apparent in the DHS, most likely due to the exclusion of the most insecure survey clusters from its sampling frame.

**Conclusion:**

MPS offers a viable alternative or complement to traditional face‐to‐face surveys for estimating child mortality in populations where insecurity limits face‐to‐face survey data collection.

## 1 Introduction

For more than a decade, several Sahelian countries have been plagued by terrorist attacks, organised crime and armed conflict between Islamist groups and government forces. Burkina Faso is no exception, and since the 2015 coup d'état, the situation has deteriorated significantly. According to the Armed Conflict Location and Event Data (ACLED) project, about one in five people in the country were exposed to armed violence within a 5 km radius in 2024 [1]. More than 2 million people (around 10% of the population) have fled their homes and around 40% of the national territory is currently beyond the control of the government [2, 3, 4]. This widespread insecurity is likely to have both direct and indirect effects on the health and survival of the affected populations, but these are not systematically documented due to the incompleteness of death registration and the limitations of the health information system [5]. In this contribution, we assess whether some of that information gap can be filled using mobile phone survey (MPS) estimates of under‐five mortality.

In settings without complete death registration, under‐five mortality estimates are typically derived from population censuses and household surveys. During such inquiries, data on mortality are collected during in‐person (or face‐to‐face) interviews. Selected respondents are asked to report recent deaths among members of their households and families [6]. Demographic and Health Survey (DHS) estimates for Burkina Faso suggest that under‐five mortality declined in recent years, despite the ongoing security crisis: From 129 per 1000 in the 5 years prior to the 2010 DHS to 48 per 1000 in the period leading up to the 2021 DHS. Estimates produced by the United Nations Inter‐Agency Group for Child Mortality Estimation (UN IGME) heavily rely on household survey data and are also suggestive of this downward trend [7]. The latest Burkina Faso DHS was, however, excluded from UN IGME due to data quality concerns. In the 2021 DHS, only 5 of the 13 regions were fully surveyed. In the Sahel, the region worst‐affected by insecurity, 28 of the 41 clusters were excluded from the sample due to security concerns (Figure 1). If these excluded areas experienced excess mortality relative to the rest of the country, for example directly due to conflict‐related deaths, or indirectly due to more difficult access to healthcare services, then a household survey might misrepresent the true level of under‐five mortality in Burkina Faso. The DHS survey report specifies that the results for the Eastern and Sahel regions should be interpreted with caution [8].

**FIGURE 1:**
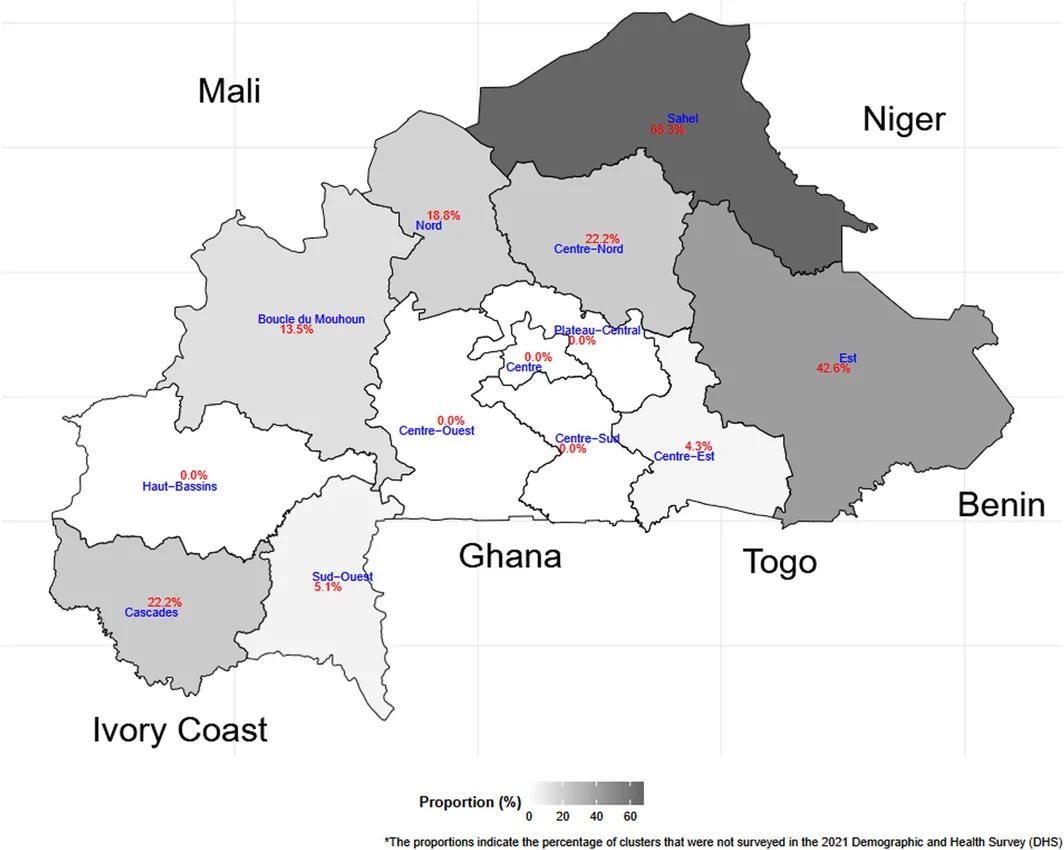
Geographic distribution of non‐surveyed clusters during the 2021 DHS in Burkina Faso.

There is thus a need for alternative data collection methods whenever face‐to‐face surveys are not feasible. Owing to the rapidly expanding mobile phone networks and ownership in many low and middle‐income countries (LMICs), MPS have become an increasingly viable modality for collecting health and mortality data [9, 10, 11]. In Burkina Faso, according to the 2019 census, the proportion of adults who personally own a mobile phone was 85% [12]. The 2021 DHS reports 75% and 90% ownership among women and men of reproductive age, respectively [8].

The Ebola and COVID‐19 outbreaks accelerated the use of mobile phones in data collection [13, 14, 15, 16, 17, 18, 19, 20, 21, 22], but mortality estimation from MPS data remains relatively rare [17, 18]. Few studies have formally evaluated how they can be used to complement or substitute for face‐to‐face surveys in populations affected by security crises, exempt one study, conducted in Northern Cameroon that compared mortality rates derived from phone interviews with those from face‐to‐face surveys [19]. Most studies of mortality in conflict‐affected settings, including the aforementioned study from Cameroon, rely on reports of recent household deaths, and there is a need to understand which other instruments, such as birth or pregnancy histories (for child mortality) and sibling histories (for adult mortality) can be used or adapted for MPS.

There are several methodological challenges associated with estimating mortality from MPS, including coverage error due to inequities in mobile phone ownership and other forms of selection bias emanating from the low response rates. The latter may be exacerbated in settings with armed conflict because respondents may be more hesitant to engage with interviewers over the phone or due to the degradation of the telecommunications infrastructure in conflict zones. While careful sampling procedures and post‐stratification weighting can alleviate bias [20], the effectiveness of weighting is likely to be context‐specific, dependent on the indicator of interest and the weighting procedure that is used [20, 21, 22]. In the absence of a gold standard measurement, it is not possible to establish whether our approach fully corrects selection bias, and we merely compare to the Burkina Faso DHS to highlight differences in the under‐five mortality estimates from MPS and face‐to‐face surveys.

In this study, we use data from the Burkina Faso Rapid Mortality MPS (RaMMPS), which combines two MPS designs: Random digit dialling (RDD) and follow‐up interviews with households previously surveyed through face‐to‐face interviews in a nationally representative household survey. We compare under‐five mortality estimates derived from the Burkina Faso RaMMPS with those from the 2021 DHS, with particular attention to differences across security contexts and time periods. This study thus contributes to the growing methodological literature on the use of MPS for mortality measurement in fragile and conflict‐affected settings.

### 1.1 The Burkina Faso Context

#### 1.1.1 Security Context and Its Impacts in Burkina Faso (2015–Present)

The collapse of the Libyan regime in 2011 and ongoing instability in Mali fed an armed insurgency in Burkina Faso, which is responsible for numerous deaths and widespread population displacement [23, 24]. The insurgency began with sporadic attacks in 2015, but gradually escalated, disproportionately affecting border regions such as the Sahel, North, East and Boucle du Mouhoun, as well as the Center‐North and Center‐East regions [25]. Approximately 40% of the country's territory is now considered beyond the control of the government [26].

The deterioration of the security situation, measured by the number of direct insurgent‐related deaths in each region, is shown in Figure 2 [1]. In 2018, most fatalities resulted from attacks on military targets, but by 2019, civilians had become the primary victims [1]. That same year, a state of emergency was declared in 14 provinces. The violence intensified in 2020, with a surge in attacks targeting civilians, humanitarian aid programmes and local self‐defence groups. A temporary lull occurred between November 2020 and January 2021 due to a negotiated ceasefire during the presidential and legislative elections. In 2022, insurgent attacks led to a sharp increase in fatalities.

**FIGURE 2:**
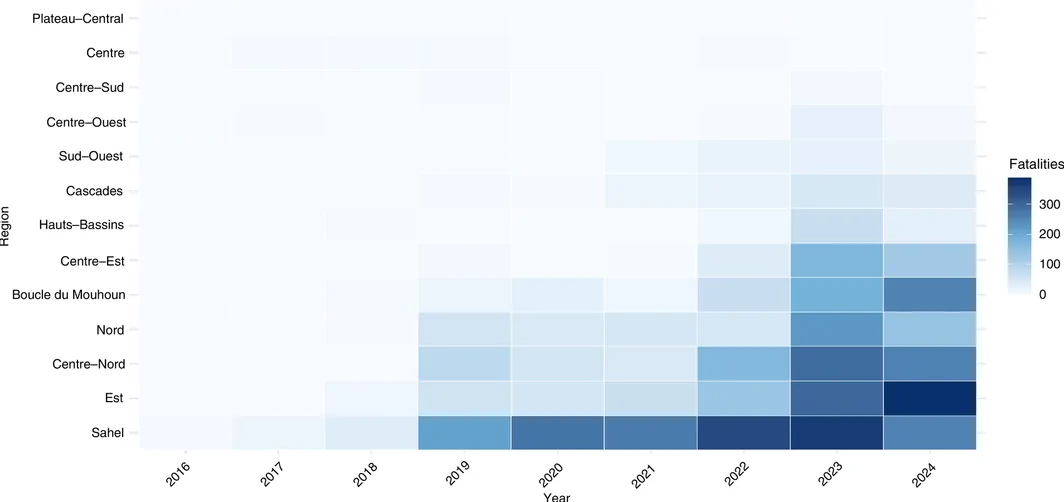
Violence‐related fatalities by region in Burkina Faso 2016–2024.

The consequences of this crisis are far‐reaching. Nearly 10% of the population has been displaced, exacerbating food insecurity and social tensions. Agriculture, which provides a livelihood for 80% of the Burkinabe population, has been severely impacted by land loss and blockades imposed by armed groups, pushing the number of food‐insecure individuals over 2.3 million in 2022 [27]. Healthcare services, already fragile, have been severely disrupted [28]. According to the Ministry of Health, more than 38% of healthcare facilities are affected, restricting access to medical care for 2.5 million individuals [29].

## 2 Data and Methods

### 2.1 Data

This study draws on data from the Burkina Faso RaMMPS along with data from the 2019 population and housing census and the 2021 DHS. The Burkina Faso RaMMPS survey was implemented by the Institut Supérieur des Sciences de la Population (ISSP) of Joseph Ki‐Zerbo University, and targeted respondents aged 15 and above. Data collection took place through quarterly cross‐sectional surveys from September 2021 to October 2022. Two sampling methods were used as follows: (i) A sample of individuals who had previously participated in the face‐to‐face Harmonized Survey on Household Living Conditions (in French EHCVM: Enquête Harmonisée sur les Conditions de Vie des Ménages) in 2018–2019 [30] and (ii) a sample of individuals obtained through RDD. A third‐party service provider (Viamo) screened randomly generated phone numbers to ensure that they were active and reachable on the mobile network. As explained elsewhere, the RDD sample used quotas for population strata in terms of place of residence, age group and sex [31]. Quotas were derived from the population distribution on these attributes in the 2019 Burkina Faso census [12]. To increase statistical power, most of the results presented in this study pool data from both samples.

Respondents provided verbal informed consent and consenting respondents completed a ±20 min survey on household and individual background characteristics, mortality among household members and close relatives (including children) and COVID‐19 vaccination. Interviewers were trained to conduct interviews with the necessary empathy, emphasise voluntary participation and the right to refuse or stop at any time. The study protocol was approved by the Observational Research Ethics Committee of the London School of Hygiene and Tropical Medicine (reference: 26393/RR/24486) and the Burkina Faso Health Ethics Committee (Ref N° 2021–07–177).

### 2.2 Methods

#### 2.2.1 Post‐Stratification Weighting

The sample of MPS respondents differed from the latest census in terms of a number of background characteristics (see Table SI‐2.1). These differences can be attributed to inequalities in mobile phone ownership and selective non‐response. According to DHS data, approximately 90% of urban women own a mobile phone, compared with about 68% of rural women. Similarly, disparities are observed in terms of wealth (from roughly 51% among women in the lowest wealth quintile to about 91% in the highest quintile), education (from 71% among women without formal education to nearly 99% among women with higher education) and region (from 38% in the Sahel region to 90% in the Central region including Ouagadougou) [32]. Such disparities imply unequal probabilities of inclusion MPS and are only partially resolved by imposing quotas in the RDD arm. The MPS sample was therefore weighted using raking weights to ensure that the marginal distributions of the weighted sample matched the census in terms of age, type of place of residence, household assets, education and region (conflict vs. non‐conflict affected regions) (further details are provided in [9]). In addition, we calculated a second set of weights specifically for women of reproductive age to ensure that the weighted distribution of women aged 15–49, aligned with the census. The combined weighting for the general population and targeted adjustment for reproductive‐age women helps to ensure that both the overall sample and the sub‐population of interest accurately reflect census distributions (Tables 1 and Table SI‐2.1). Post‐stratification weighted estimates are presented in the main text, while results from sensitivity analyses using alternative weighting strategies are reported in the SI‐3 in Supporting Information.

**TABLE 1:** Comparison of women's characteristics according to data collection source (DHS, MPS and the census).

	MPS			DHS		Census %
	Unweighted	Weighted		Weighted		
	%	%	95% CI	%	95% CI	
Age group						
15–29 years	49.1	58.5	[57.3–59.7]	55.3	[54.5–56.1]	59.0
30–49 years	50.9	41.5	[40.3–42.7]	44.7	[43.9–45.5]	41.0
Education						
None	47.0	67.1	[65.9–68.2]	58.3	[57.5–59.1]	67.4
Primary	15.6	22.7	[21.7–23.8]	14.2	[13.7–14.8]	22.8
Secondary and higher	37.4	10.2	[9.6–10.9]	27.5	[26.7–28.2]	9.7
Marital status						
Married	76.8	81.1	[80.1–82.0]	72.9	[72.1–73.6]	70.3
Widowed	2.6	3.4	[3.0–3.8]	1.6	[1.4–1.8]	2.0
Divorced/sep	1.0	0.9	[0.7–1.1]	1.8	[1.6–2.0]	0.9
Single	19.6	14.7	[13.9–15.6]	23.8	[23.1–24.5]	26.8
Place of residence						
Urban	42.1	29.9	[28.8–31.0]	32.5	[31.7–33.3]	29.9
Rural	57.9	70.1	[69.0–71.2]	67.5	[66.7–68.3]	70.1
Household size						
1–4 pers	21.7	22.4	[21.4–23.4]	18.5	[17.8–19.1]	29.9
5–8 pers	44.9	40.6	[39.4–41.8]	44.0	[43.2–44.9]	40.6
9 pers.&+	33.3	37.0	[35.8–38.2]	37.5	[36.7–38.3]	29.5
HH has electricity^a^						
Yes	75.2	55.9	[54.7–57.2]	24.1	[23.3–24.8]	56.0
No	24.8	44.1	[42.8–45.3]	75.9	[75.2–76.7]	44.0
Dwelling has durable roofing material						
Yes	91.0	84.6	[83.7–85.5]	87.0	[86.4–87.6]	81.2
No	9.0	15.4	[14.5–16.3]	13.0	[12.4–13.6]	18.8
Household has access to an improved source of drinking water						
Yes	86.7	78.7	[77.7–79.7]	31.4	[30.6–32.2]	68.5
No	13.3	21.3	[20.3–22.3]	68.6	[67.8–69.4]	31.5

*Note:* The weighting scheme used in this study differs from that used in a previous publication based on the same survey [31]. Here, survey weights were recalibrated through post‐stratification so that the distributions in women of reproductive age in the MPS match those from the census data.

^a^ The definition of household electricity access is consistent between the MPS and the census. However, for the DHS, the exact construction of the variable is less clear because detailed information is not available.

#### 2.2.2 Mortality Estimation

Child mortality was estimated using a life‐table or synthetic cohort approach using data collected via truncated birth histories (TBH) reported by women of reproductive age and referring to all births that had occurred in the 7 years prior to data collection. We calculated mortality rates directly, using dates of birth and death (or date of interview for living children) to tabulate deaths and exposure time. Period rates were converted to probabilities and chained together to obtain the under‐five mortality rate (_5_q_0_) [6]. Using the same life‐table framework and age‐specific probabilities, we also estimated infant (_1_q_0_) and child mortality (_4_q_1_). This was done with the demogsurv package in the R statistical software, which allows for complex survey designs and accommodates weighting [33]. This package provides point estimates of age‐specific mortality rates along with their standard errors and confidence intervals, obtained here through Taylor linearization [33]. As a sensitivity analysis, confidence intervals were also computed using a nonparametric bootstrap approach. For each stratum, the total number of interviews was resampled 1000 times with replacement, with resampling probabilities proportional to the survey weights. For each bootstrap replicate, mortality estimates were recalculated using the same life‐table framework. The median of the bootstrap distribution was reported as the point estimate and the 2.5th and 97.5th percentiles were used to construct 95% confidence intervals. Bootstrap confidence intervals were compared with Taylor linearization derived intervals to assess the robustness of the results. Confidence intervals obtained from the Taylor linearization method closely match those derived from bootstrap resampling (SI‐1 in Supporting Information).

Mortality estimates were disaggregated by period and region. The selected periods correspond to low or no insecurity (2014–2017), moderate insecurity (2018–2019) and high insecurity (2020–2022). This classification is based on the number of violence‐related fatalities reported by the ACLED project (Figure 2). A dichotomous region variable is used to distinguish regions that are affected by conflict (i.e., Sahel, North, Center‐North, Boucle du Mouhoun, Center‐East and East) and regions that are more secure. Again, this classification is based on the number of violence‐related fatalities reported in Figure 2. To assess whether observed differences across groups and over time were statistically meaningful, we compared mortality estimates using their 95% confidence intervals.

## 3 Results

### 3.1 Characteristics of Study Participants

A total of 52,650 phone numbers were tried in the Burkina Faso RaMMPS, resulting in 11,387 interviews with women of reproductive age. Of these, 8077 were reached through RDD (36% lived in conflict‐affected areas) and 3310 via previously collected EHCVM numbers (42% lived in conflict‐affected areas). Table SI‐2.2 summarises the phone call outcomes following guidelines from the American Association for Public Opinion Research (AAPOR) [34]. Response, cooperation and contact rates were all higher in the EHCVM arm, which recontacted respondents previously interviewed face‐to‐face, compared to the RDD approach. On average, 2 phone numbers were needed per completed interview in the EHCVM arm, compared with 4 in the RDD arm. The response rate was 59% in the EHCVM arm, compared to 45% in the RDD arm. In order to establish eligibility, respondents had to answer a few questions and most eligible respondents who answered the phone agreed to participate (91% EHCVM; 84% RDD). Unfortunately, as it is not possible to determine the region of residence for respondents who did not complete the interview, the potential regional variations in non‐response cannot be explored.

Table 1 reports the comparison of characteristics of women of reproductive age across data sources, presenting both weighted and unweighted proportions for the MPS, weighted proportions for the DHS and the corresponding proportions from the census. Unweighted MPS respondents differ substantially from the census population: they are more often urban residents, wealthier, better educated and older, and these attributes are often associated with more favourable health profiles. After post‐stratification, weighted MPS distributions closely align with those of the census, although discrepancies remain for variables that were not included in the weighting procedure (e.g., household size and marital status).

Tables SI‐2.3 and SI‐2.4 provide further details by disaggregating these distributions by security context and across the two samples of the RaMMPS survey, presenting both weighted and unweighted proportions. In conflict‐affected areas, weighted distributions of background characteristics are largely comparable between the DHS and the MPS. However, the MPS sample contains a higher proportion of households with more than five members compared to the census and includes fewer women from rural areas. It is also worth noting that differences are observed in respondents' characteristics between the DHS and the census, although both data sources rely on face‐to‐face data collection. These differences may be due to variations in how the questions are phrased. According to the DHS, households are less frequently connected to electricity and piped water than recorded in the census. Additionally, discrepancies are evident in educational attainment, with the DHS reporting a higher proportion of educated individuals compared to the census. In non‐conflict areas (Table SI‐2.3), weighted MPS distributions align more closely with census benchmarks for most sociodemographic characteristics, although some residual differences remain in these regions as well (marital status and household size). The DHS continues to display higher proportions of educated women and women living in wealthier households compared to the census, even after weighting is applied.

Table SI‐2.4 highlights systematic differences between the two MPS arms. In both conflict and non‐conflict areas, the RDD sample consists of younger, more often urban and smaller households compared to the EHCVM arm. After weighting, these differences are reduced but not fully eliminated, particularly for variables not included in the post‐stratification (household size and marital status).

### 3.2 Under‐Five, Infant and Child Mortality Levels and Trends

Figure 3 presents under‐five mortality trends in conflict‐affected areas and in areas that have remained relatively secure. The under‐five mortality rate estimated from the DHS declined slightly from about 54 per 1000 in 2018–2019 (95% CI: 43–64) to 45 per 1000 in 2020–2022 (95% CI: 36–55) in conflict‐affected areas and remained stable from about 45 per 1000 in 2018–2019 (95% CI: 36–54) to 44 per 1000 in 2020–2022 (95% CI: 33–55) in more secure areas. Mortality stagnation also characterises the MPS data for regions that were less affected by armed conflict. In contrast, the MPS estimates show a clear and statistically significant mortality increase in conflict‐affected areas: from 47 per 1000 in 2018–2019 (95% CI: 38–57) to 82 in 2020–2022 (95%‐CI: 67–98).

**FIGURE 3:**
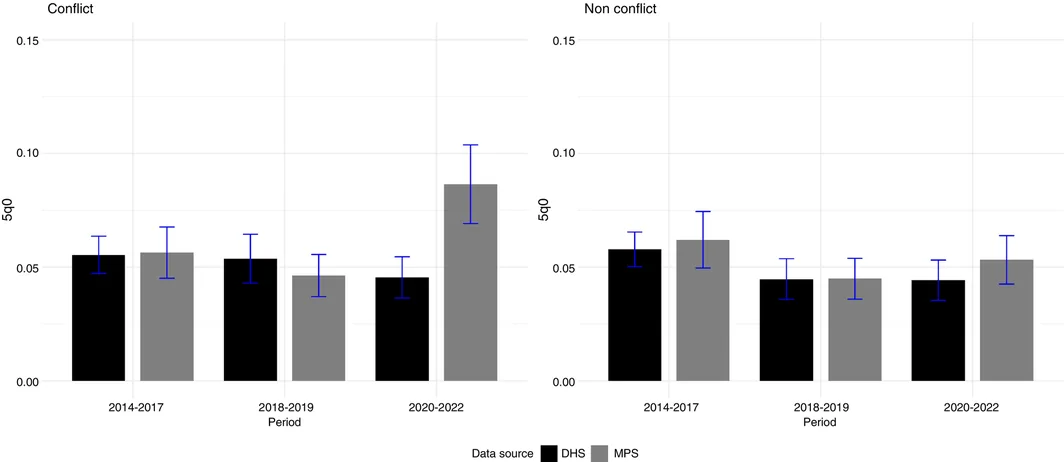
Trends in under‐five mortality based on regional security context, derived from the MPS and the 2021 DHS.

Figure 4 illustrates the levels and trends in under‐five mortality by data source, period and security status. As in Figure 3, there are no statistically significant differences in mortality levels across the various data sources for any given period in non‐conflict areas. In conflict‐affected areas, the two RaMMPS samples (EHCVM and RDD) are indicative of a mortality reversal between 2018–2019 and 2020–2022. As indicated above, this is not the case for the DHS estimates. Further, it is worth noting that the MPS mortality estimates tend to be lower for the RDD than for the EHCVM sample. For the most recent period, this difference is, however, only marginally statistically significant.

**FIGURE 4:**
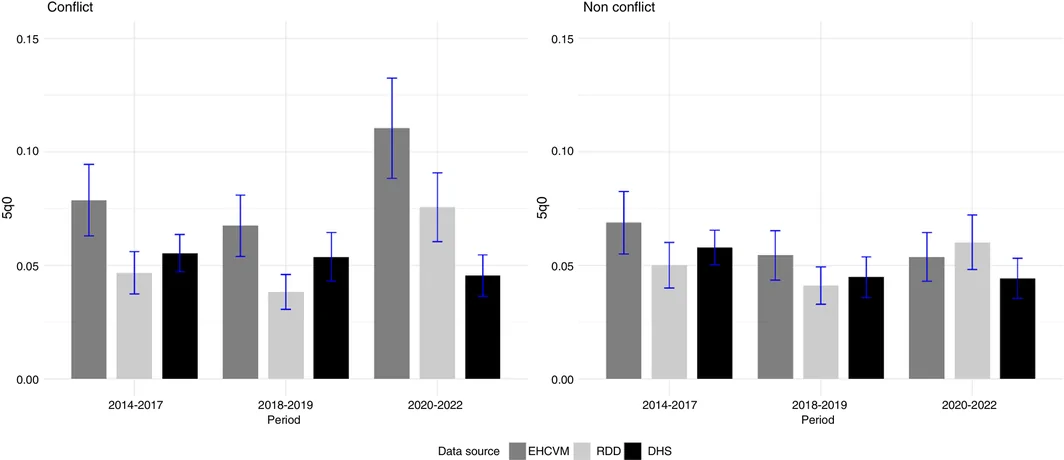
Trends in under‐five mortality based on regional security context and survey sampling method.

To ensure the robustness of our findings, we conducted sensitivity analyses by exploring alternative weighting strategies, different definitions of conflict exposure and different time periods (SI‐3 in Supporting Information). The results remained consistent across all specifications.

The patterns observed in the overall under‐five mortality estimates, including variation across RaMMPS samples, were also observed when we disaggregated the mortality estimates between infant (_1_q_0_) and child (_4_q_1_) mortality. This is illustrated in Figures SI‐4.1–SI‐4.4 in the appendix. As expected, the DHS and the MPS are indicative of higher mortality among infants compared to children aged 1 to 4. The mortality increase (2020–2022) in conflict‐affected areas is observed in both age groups in the MPS survey, but it is more pronounced among children aged 1–4. In terms of the *relative* difference between MPS and DHS levels, the gap is substantially larger for children aged 1–4 (+124.6%) than for infants (+60.3%) (see Figures SI‐4.1 and SI‐4.3).

To understand the source of the differences with the DHS, we calculated the distribution of the children mentioned in the MPS based on whether or not their province of residence was included in the DHS (Table SI‐5 in Supporting Information). Table SI‐5 in the Supporting Information shows the distribution of children in the MPS based on whether their province of residence was included in the DHS. The “Excluded” category consists of the provinces of Oudalan and Yagha in the Sahel region, as well as the provinces of Kompienga, Komondjari and Tapoa in the Eastern region. Overall, only 1.8% (*n* = 204) of the surveyed children lived in provinces not covered by the DHS, while 98.2% (*n* = 11,018) resided in provinces included in the DHS. Only a small share of children reported in birth histories were residing in provinces that were excluded from the DHS, but MPS were able to reach populations living in areas entirely omitted from face‐to‐face survey operations due to insecurity.

In terms of mortality, 5.4% (11/204) of children in provinces excluded from the DHS sample had died, compared to 5.2% (544/11,018) in included provinces. Despite this minor difference, under‐five mortality rates are significantly higher in the provinces excluded from the DHS (labelled “MPS‐Excluded”), suggesting that the DHS has underestimated the under‐five mortality rate due to the exclusion of unsafe areas from the sampling process (Figure 5). In the Sahel region, estimates from the DHS and the mobile phone interviews conducted in provinces included in the DHS sample are closely aligned. In contrast, the MPS estimates for the Eastern region remain higher than those from the DHS, even for the same provinces. This could possibly be explained by the heterogeneity of situations within the Eastern region. Although some provinces were officially classified as “secure” and included in the DHS sampling frame, security conditions often varied significantly within the same province. Consequently, the DHS survey may have only reached the accessible parts of these provinces, omitting harder‐to‐reach or partially insecure areas. In contrast, the MPS relying on mobile phone interviews could have reached respondents in these more remote or insecure localities, potentially contributing to the higher mortality estimates observed.

**FIGURE 5:**
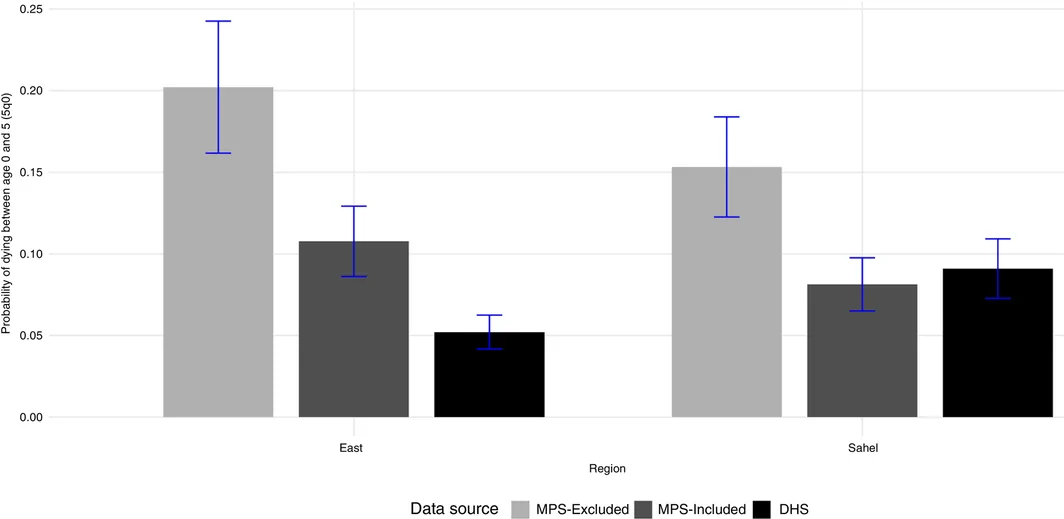
Estimated U5MR for the period 2020–22 by survey source and region of residence.

## 4 Discussion

This study assessed whether MPS might complement or remedy the limitations of in‐person data collection during humanitarian crises, using Burkina Faso as a case study. Uncertainties surrounding the funding of the DHS Program following the dismantling of the US Agency for International Development by the Trump administration further heightens the relevance of cheaper alternatives to large household surveys for monitoring demographic and health trends in LMICs [35]. We have to date limited experience with MPS for measuring mortality. A number of studies demonstrated acceptability and feasibility [36, 37]. Collecting information on the deaths of relatives by phone may seem difficult, as respondents might perceive such questions as too sensitive, particularly in insecure settings. However, evidence from a study in Malawi suggests cooperation rates were just as high for questions about recent deaths among relatives as for questions about recent economic activity. Moreover, respondents rarely reported negative feelings when asked about deceased relatives and such reactions were no more frequent than among those answering economic questions [36]. Coverage error arising from unequal mobile phone ownership and differential participation rates are other key methodological challenges that can limit the external validity of MPS. These can be mitigated through the imposition of sampling quotas and post‐stratification weighting. They do indeed offer a correction in the expected direction, but in the absence of a gold standard, it is impossible to establish whether this removes bias entirely.

Our study shows that MPS are better suited for estimating mortality in populations affected by humanitarian crises than traditional face‐to‐face surveys such as the DHS. In the most secure provinces of Burkina Faso, under‐five mortality estimates from both the MPS and the DHS are comparable and suggestive of stagnating mortality levels. In provinces where armed conflict is most pronounced, MPS are indicative of a mortality reversal (after 2019); a trend that is not captured in the 2021 Burkina Faso DHS. Our analysis suggests that this downward bias is due to the exclusion of the most inaccessible areas from the DHS sampling frame. This corroborates a study from Cameroon that highlighted the potential of MPS for mortality measurement in areas with insecurity [19]. In our study, the mortality increases in conflict‐affected areas characterised both arms of the MPS, irrespective of the sampling method. However, the respondents recruited from pre‐existing phone numbers tended to report a higher number of under‐five deaths than those in the sample constituted through RDD. It is possible that the establishment of a relationship of trust was facilitated when calling people who had previously been contacted compared to those called at random.

In areas without security challenges, we found no significant difference in mortality estimates between telephone and face‐to‐face surveys. This finding corroborates observations from other RaMMPS surveys conducted in Malawi [36] and Mozambique [17].

The elevated child mortality in areas affected by armed conflict can be attributed to several factors. First, insecurity may directly cause violent deaths, as children can be unintentionally or intentionally harmed during attacks, explosions or crossfire [1]. While these events may not represent the majority of child deaths, their occurrence increases overall mortality levels and reflects the immediate consequences of living in unstable environments. Second, the security crisis has led to the deterioration of already fragile healthcare infrastructure, limiting access to health services for millions of displaced and non‐displaced individuals [5, 26, 38]. Further, the economic slowdown or cessation of activities in high‐conflict areas has exacerbated financial strain [39, 40, 41, 42] and worsened child malnutrition [43, 44]. These security‐related challenges were compounded by the COVID‐19 crisis [45, 46]. For internally displaced persons in particular, COVID‐19 worsened living conditions, reducing humanitarian aid and increasing malnutrition [47]. Research also highlighted difficulties in accessing healthcare services during the pandemic, even in rural areas [48]. Additional studies have highlighted the combined negative effects of COVID‐19 and insecurity on people's livelihoods, notably by disrupting agricultural activities, exacerbating psychosocial distress among displaced populations and limiting access to essential services [39, 49]. These negative impacts increase the risk of mortality in the general population, particularly among children. Although infants typically experience higher mortality levels than children aged 1–4 years, a trend well documented in low‐and‐middle income countries [50, 51], our results suggest that the increase in mortality was more pronounced above age 1. The *relative* difference between MPS and DHS estimates was also greater for child mortality (_4_q_1_) than for infant mortality (_1_q_0_) in conflict‐affected areas. This pattern indicates that the adverse effects of insecurity may weigh more heavily on children aged 1–4 than on infants. Infants depend heavily on maternal care, including breastfeeding, which helps protect them from infections and nutritional deficiencies even in unstable environments [52]. In contrast, children aged 1–4 are more susceptible to environmental risks as household food security becomes more important to prevent malnutrition and they become more mobile in unsafe surroundings. These factors increase their risk of malnutrition, diarrheal diseases, respiratory infections and decreased care‐seeking during crises, issues known to elevate under‐five mortality during conflicts [53].

While our findings demonstrate the potential of MPS for measuring mortality in crisis‐affected settings, it is crucial to maintain a cautious perspective. Several unresolved issues remain. First, challenges related to data quality persist, including potential reporting errors and recall biases in MPS that are not yet fully understood. Second, it is important to acknowledge some limitations related to the survey instruments used in the RaMMPS. We did not, for example, collect specific information on households' exposure to violence or forced displacement. Further, we used the truncated birth history (TBH) instrument in the Burkina Faso RaMMPS so as to limit the interview duration, but it tends to produce downward biased estimates of under‐five mortality [6, 54].

## 5 Conclusion

MPS provides a viable complement to traditional surveys for estimating child mortality, particularly in insecure regions where conventional methods are impractical. MPS can effectively capture critical mortality trends in high‐risk areas that face‐to‐face surveys may not cover. Our results highlight the necessity for further research into potential biases related to data collection, sample representativeness and the exclusion of high‐risk areas during fieldwork in insecure regions. Policymakers should consider integrating MPS into national data collection strategies to ensure comprehensive and timely information, especially during health and security crises.

## Funding

This study was made possible with financial support from the *Bill and Melinda Gates Foundation* (INV‐023211). The funder had no role in study design, data collection, data analysis, data interpretation or writing of the report.

## Ethics Statement

The study protocol was reviewed by the Ethics Committee of the London School of Hygiene and Tropical Medicine (reference: 26393/RR/24486) and the Ethics Committee of the Ministry of Health of Burkina Faso. All participants provided oral consent for the survey, including consent for storing anonymised data in a public repository and consent to audio record the interview.

## Conflicts of Interest

The authors declare no conflicts of interest.

## Supporting information


**Table SI‐1.1:** Under‐five mortality estimates by source, region and period.
**Table SI‐1.2:** Under‐five mortality estimates by source, region and period.
**Table SI‐1.3:** Under‐five mortality estimates by source, region and period.
**Table SI‐1.4:** Under‐five mortality estimates by source, region and period.
**Table SI‐2.1:** Composition of the MPS sample, by study arm and comparison with characteristics of the population enumerated in the 2019–2020 census.
**Table SI‐2.2:** Final disposition codes and phone call outcome rates by study arm.
**Table SI‐2.3:** Comparison of women's characteristics by regional context of their place of residence in the MPS, DHS and the census.
**Table SI‐2.4:** Comparison of women's characteristics according to the regional context of their place of residence using RaMMPS data, by study arm.
**Table SI‐3.1:** Characteristics of respondents by phone ownership.
**Table SI‐3.2:** Under‐five mortality (5q0) by period, region, data source and weighting method.
**Figure SI‐3.1:** Sensitivity of under‐five mortality estimates across data sources, conflict levels and periods.
**Figure SI‐3.2:** Sensitivity of under‐five mortality estimates across data sources, conflict levels and periods.
**Figure SI‐4.1:** Trends in child mortality (4q1) based on regional security context, derived from RaMMPS survey and 2021 DHS.
**Figure SI‐4.2:** Trends in child mortality (4q1) based on regional security context and survey sampling method.
**Figure SI‐4.3:** Trends in infant mortality (1q0) based on regional security context, derived from RaMMPS survey and 2021 DHS.
**Figure SI‐4.4:** Trends in infant mortality (1q0) based on regional security context and survey sampling method.
**Table SI‐5:** Percentage of surveyed and deceased children in MPS sample by inclusion in the DHS sampling frame.

## Data Availability

The data that support the findings of this study are available on request from the corresponding author and Bruno Lankoande (blankoande@issp.bf). The data are not publicly available due to privacy or ethical restrictions.

## References

[1] ACLED , “Conflict Exposure: distribution of the total number of deaths from insurgent attacks per month by target type. ACLED [Internet],” (2025), https://acleddata.com/conflict-exposure/.

[2] UNHCR A. UNHCR Africa [Internet] , “Burkina Faso,” (2025), https://www.unhcr.org/africa/countries/burkina-faso.

[3] Human Rights Watch , “Burkina Faso: Événements de 2022. In: Rapport mondial 2023 [Internet],” (2023), https://www.hrw.org/fr/world-report/2023/country-chapters/383648.

[4] WFP , “Burkina Faso: Mise à jour de la situation, Mai 2023—Burkina Faso|ReliefWeb [Internet],” (2023), https://reliefweb.int/report/burkina-faso/burkina-faso-mise-jour-de-la-situation-mai-2023.

[5] O. Ouedraogo , S. Siguré , S. Sanou , A. Kaboré , M. M. Dahany , and S. Diallo , “Landscape Analysis of Healthcare Workforce and Resilience Actions in the Sahelian Context of High Security Challenge Areas: The Case of Burkina Faso,” International Journal of Health Planning and Management 39, no. 3 (2024): 933–944, 10.1002/hpm.3764. 38240163

[6] K. Hill , “Direct Estimation of Child Mortality From Birth,” in Tools for Demographic Estimation [Internet], ed. T. Moultrie , R. Dorrington , A. Hill , K. Hill , I. Timæus , and Z. Zaba (UFPA, 2014).

[7] UN IGME , Levels and Trends in Child Mortality: Report 2025 (United Nations Inter‐Agency Group for Child Mortality Estimation, 2026), https://www.childmortality.org/.

[8] INSD , “Enquête Démographique et de Santé 2021 [Internet],” (2021), https://dhsprogram.com/pubs/pdf/OF49/OF49pou.pdf.

[9] L. Ben , M. Robert , M. Jonathan , P. Tiago , and D. Stephen , “Do Mobile Phone Surveys Work in Poor Countries? ‐ Working Paper 398|Center for Global Development|Ideas to Action [Internet],” (2015), https://www.cgdev.org/publication/do-mobile-phone-surveys-work-poor-countries-working-paper-398.

[10] D. G. Gibson , A. Pereira , B. A. Farrenkopf , A. B. Labrique , G. W. Pariyo , and A. A. Hyder , “Mobile Phone Surveys for Collecting Population‐Level Estimates in Low‐ and Middle‐Income Countries: A Literature Review,” Journal of Medical Internet Research 19, no. 5 (2017): e139, 10.2196/jmir.7428. PMC5438460 28476725

[11] A. Labrique , E. Blynn , S. Ahmed , D. Gibson , G. Pariyo , and A. A. Hyder , “Health Surveys Using Mobile Phones in Developing Countries: Automated Active Strata Monitoring and Other Statistical Considerations for Improving Precision and Reducing Biases,” Journal of Medical Internet Research 19, no. 5 (2017): e7329, 10.2196/jmir.7329. PMC5438457 28476726

[12] INSD , “Résultats du 5E Recensement Général de la Population et de l'Habitation|INSD [Internet],” (2019), https://www.insd.bf/fr/resultats.

[13] C. Lamanna , K. Hachhethu , S. Chesterman , et al., “Strengths and Limitations of Computer Assisted Telephone Interviews (CATI) for Nutrition Data Collection in Rural Kenya,” PLoS One 14, no. 1 (2019): e0210050, 10.1371/journal.pone.0210050. PMC6353544 30699207

[14] PMA , “PMA Burkina Faso COVID‐19 Dashboard French|PMA Data [Internet],” (2023), https://www.pmadata.org/pma-burkina-faso-covid-19-dashboard-french.

[15] M. Vinceti , T. Filippini , K. J. Rothman , et al., “Lockdown Timing and Efficacy in Controlling COVID‐19 Using Mobile Phone Tracking,” EClinicalMedicine 25 (2020): 100457, 10.1016/j.eclinm.2020.100457. PMC7355328 32838234

[16] World Bank Group , “Monitoring COVID‐19 Impacts on Firms in Ethiopia : Results from a High‐Frequency Phone Survey of Firms,” (2020), https://documents.worldbank.org/en/publication/documents-reports/documentdetail/515671597673928629/Monitoring-COVID-19-Impacts-on-Firms-in-Ethiopia-Results-from-a-High-Frequency-Phone-Survey-of-Firms.

[17] A. M. Kante , C. Manhica , A. Aveika , et al., “Measuring Childhood Mortality Through Mobile Phone Interviews in Mozambique,” Tropical Medicine and Interational Health 30 (2025): 1087–1096, 10.1111/tmi.70004. PMC12501560 40888227

[18] G. Reniers , J. Romero‐Prieto , M. Chasukwa , et al., “Mobile Phone Survey Estimates of Perinatal Mortality in Malawi: A Comparison of Data From Truncated and Full Pregnancy Histories,” Tropical Medicine and Interational Health 30, no. 6 (2025): 521–530, 10.1111/tmi.14109. PMC12136939 40254889

[19] E. M. H. Gignoux , O. T. Donfack Sontsa , A. Mudasiru , et al., “A Telephone Based Assessment of the Health Situation in the Far North Region of Cameroon,” Conflict and Health 14, no. 1 (2020): 82, 10.1186/s13031-020-00327-4. PMC7708193 33292396

[20] D. A. Sánchez‐Páez , B. Masquelier , A. Menashe‐Oren , O. J. Baruwa , and G. Reniers , “Measuring Under‐5 Mortality and Fertility Through Mobile Phone Surveys: An Assessment of Selection Bias in 34 Low‐Income and Middle‐Income Countries,” BMJ Open 13, no. 11 (2023): e071791, 10.1136/bmjopen-2023-071791. PMC10693685 37977863

[21] A. R. Greenleaf , A. Gadiaga , G. Guiella , et al., “Comparability of Modern Contraceptive Use Estimates Between a Face‐To‐Face Survey and a Cellphone Survey Among Women in Burkina Faso,” PLoS One 15, no. 5 (2020): e0231819, 10.1371/journal.pone.0231819. PMC7219703 32401773

[22] A. Sahar , P. Julio , S. David , B. Masquelier , T. W. Pullum , and G. Reniers , “Sample Selection Bias in Adult Mortality Estimates From Mobile Phone Surveys: Evidence From 25 Low‐ and Middle Income Countries,” Demographic Research 51 (2024): 1167–1182.

[23] B. K. Moon , “Letter Dated 17 January 2012 From the Secretary‐General Addressed to the President of the Security Council,” Journal of International Peacekeeping 6, no. 4–6 (2012): 219–221, 10.1163/187541100X00319.

[24] Action the Center for Preventive. Global Conflict Tracker [Internet] , “Violent Extremism in the Sahel,” (2025), https://cfr.org/global-conflict-tracker/conflict/violent-extremism-sahel.

[25] W. Renault , Le Burkina Faso Face à la Menace Djihadiste. Portail de L'IE [Internet], (2019), https://www.portail-ie.fr/univers/surete-et-gouvernance-des-risques/2019/le-burkina-faso-face-a-la-menace-djihadiste/.

[26] HRW , “Burkina Faso: Upsurge in Atrocities by Islamist Armed Groups|Human Rights Watch [Internet],” (2023), https://www.hrw.org/news/2023/06/15/burkina-faso-upsurge-atrocities-islamist-armed-groups.

[27] OCHA , “Burkina Faso: Situation des personnes déplacées internes (PDI) (30 juin 2022)|OCHA [Internet],” (2022), https://www.unocha.org/publications/report/burkina-faso/burkina-faso-situation-des-personnes-deplacees-internes-pdi-30-juin-2022.

[28] Organisation Mondiale de la Santé , “Rapport de situation N° 21: Réponse sanitaire à la crise humanitaire au Burkina Faso,” (2023).

[29] Ministère de la Santé , “Annuaire Statistique du Ministère de la Santé du Burkina Faso” (2022).

[30] Institut National de la Statistique et de la Démographie (INSD) , Enquête Harmonisée Sur le Conditions de Vie Des Ménages 2018–2019 [Internet] (World Bank, Development Data Group, 2022), 10.48529/WV88-J486.

[31] K. Dianou , B. Masquelier , A. Soura , et al., “The Use of Mobile Phone Surveys for Rapid Mortality Monitoring: A National Study in Burkina Faso,” Demographic Research 52, no. 16 (2025): 479–518.

[32] DHS Program , “STATcompiler [Internet],” (2026), https://www.statcompiler.com/fr/.

[33] J. W. Eaton and B. Masquelier , “Demogsurv: Demographic Analysis of DHS and Other Household Surveys [Internet],” (2021), https://mrc-ide.r-universe.dev/demogsurv.

[34] AAPOR , “Standard Definitions: Final Dispositions of Case Codes and Outcome Rates for Surveys 9th edition,” (2016).

[35] G. Duthé , “Arrêt de L'USAID: Un Grand Frein Aux Recherches Sur la Santé et le Développement—AOC Media‐Analyse Opinion Critique [Internet],”.

[36] M. Chasukwa , A. T. Choko , F. Muthema , et al., “Collecting Mortality Data via Mobile Phone Surveys: A Non‐Inferiority Randomized Trial in Malawi,” PLOS Global Public Health 2, no. 8 (2022): e0000852, 10.1371/journal.pgph.0000852. PMC10021539 36962430

[37] R. Morgan , Y. Manganhe , C. Monjane , et al., “Women's Participation in Mobile Phone Surveys in Mozambique: Findings From a Qualitative Study,” Tropical Medicine & International Health 30 (2025): 946–953, 10.1111/tmi.70006. PMC12401626 40682465

[38] M. Diamondstein and Center for Reproductive Rights [Internet] , Impact of the COVID‐19 Pandemic on Sexual and Reproductive Health Services in Burkina Faso (Ethiopia, 2022).

[39] M. Badini , M. Guira , A. Guissou , A. Lhomme , and ALIMENTERRE [Internet] , “Au Burkina Faso, les Maraîchers du Bam face aux Conditions D'insécurité,”.

[40] S. Louart , E. Bonnet , and V. Ridde , “Is Patient Navigation a Solution to the Problem of « Leaving no One Behind »? A Scoping Review of Evidence From Low‐Income Countries,” Health Policy and Planning 36, no. 1 (2021): 101–116, 10.1093/heapol/czaa093. PMC7938515 33212491

[41] P. R. Nikiema and F. A. Dedewanou , “The Impact of COVID‐19 on Food Insecurity in Burkina Faso [Internet],” (2024), https://publication.aercafricalibrary.org/handle/123456789/3693.

[42] A. Tapsoba , D. A. Wardell , M. Zida , and M. Elias , “Insécurité et COVID‐19 Au Burkina Faso: Opportunités et vulnérabilités Des Femmes de la chaîne de Valeurs du karité [Internet],” (2021), https://hdl.handle.net/10568/116775.

[43] M. Costanza and IMPACT Initiatives [Internet] , Dégradation Des Conditions Sanitaires et Alimentaires Au Burkina Faso : Des Besoins Urgents Dans Une Crise oubliée|Impact (2024), https://www.impact-initiatives.org/what-we-do/news/degradation-des-conditions-sanitaires-et-alimentaires-au-burkina-faso-des-besoins-urgents-dans-une-crise-oubliee/.

[44] G. Druhen and UNICEF [Internet] , “Burkina Faso: L'insécurité Plonge Les Enfants Dans la Malnutrition,” (2023), https://www.unicef.fr/article/linsecurite-plonge-les-enfants-du-burkina-faso-dans-la-malnutrition/.

[45] A. Lanau , T. Grieve , A. F. Tengbeh , L. Enria , and M. Wayack‐Pambé , ““Since the Market Is Closed, There Is no More Money, There Is Nothing we Can Do”: Voices of Adolescent Girls in Burkina Faso and Sierra Leone on Poverty and COVID‐19,” Children and Youth Services Review 155 (2023): 107232, 10.1016/j.childyouth.2023.107232.

[46] O. Yankey , M. Essah , and P. M. Amegbor , “The COVID‐19 Pandemic and Self‐Reported Food Insecurity Among Women in Burkina Faso: Evidence From the Performance Monitoring for Action (PMA) COVID‐19 Survey Data,” BMC Women's Health 25, no. 1 (2025): 42, 10.1186/s12905-025-03565-x. PMC11786433 39893400

[47] P. Ozer , A. Dembele , S. S. Yameogo , E. Hut , and F. de Longueville , “The Impact of COVID‐19 on the Living and Survival Conditions of Internally Displaced Persons in Burkina Faso,” World Development Perspectives 25 (2022): 100393, 10.1016/j.wdp.2022.100393. PMC8743390 35036662

[48] E. Bonnet , Y. Beaugé , M. F. Ba , S. Sidibé , M. De Allegri , and V. Ridde , “Correction: Knowledge of COVID‐19 and the Impact on Indigents' Access to Healthcare in Burkina Faso,” International Journal for Equity in Health 22, no. 1 (2023): 10, 10.1186/s12939-022-01797-z. PMC9835024 36635721

[49] S. Yougbaré , A. N. Ouedraogo , and Rouamba/Ouedraogo BVC , “COVID‐19 Among Internally Displaced Persons in Burkina Faso: Psychosocial Impact and Intervention Strategies,” Social Sciences Févr 11, no. 1 (2022): 1, 10.11648/j.ss.20221101.15.

[50] J. E. Lawn , S. Cousens , and J. Zupan , “4 Million Neonatal Deaths: When? Where? Why?,” Lancet 365, no. 9462 (2005): 891–900, 10.1016/S0140-6736(05)71048-5. 15752534

[51] L. Liu , S. Oza , D. Hogan , et al., “Global, Regional, and National Causes of Under‐5 Mortality in 2000–15: An Updated Systematic Analysis With Implications for the Sustainable Development Goals,” Lancet 388, no. 10063 (2016): 3027–3035, 10.1016/S0140-6736(16)31593-8. PMC5161777 27839855

[52] C. G. Victora , R. Bahl , A. J. D. Barros , et al., “Breastfeeding in the 21st Century: Epidemiology, Mechanisms, and Lifelong Effect,” Lancet 387, no. 10017 (2016): 475–490, 10.1016/S0140-6736(15)01024-7. 26869575

[53] Z. Wagner , S. Heft‐Neal , Z. A. Bhutta , R. E. Black , M. Burke , and E. Bendavid , “Armed Conflict and Child Mortality in Africa: A Geospatial Analysis,” Lancet 392, no. 10150 (2018): 857–865, 10.1016/S0140-6736(18)31437-5. PMC6338336 30173907

[54] B. Masquelier , A. Menashe‐Oren , and G. Reniers , “An Evaluation of Truncated Birth Histories for the Rapid Measurement of Fertility and Child Survival,” Population Health Metrics 21, no. 1 (2023): 8, 10.1186/s12963-023-00307-9. PMC10354946 37464429

