# Supplementary material for: Under‐Five Mortality in Humanitarian Emergencies: A Comparison of Face‐to‐Face and Mobile Phone Survey Estimates From Burkina Faso
Source: Trop Med Int Health. 2026 Jun 11;31(10):1370–80. doi: 10.1111/tmi.70176 (PMC13628443; doi:10.1111/tmi.70176)
Supplement: Supplementary file 1 [file TMI-31-1370-s001.docx]

**Supporting Information**

## SI-1: Robustness of under-Five mortality estimates: comparison of the Taylor linearization and bootstrap approaches across data sources, conflict contexts, and periods

This section presents supplementary tables comparing analytical and bootstrap estimates of under-five mortality across data sources (DHS and MPS), conflict intensity levels, regions, and time periods, in order to assess the robustness of mortality estimates to alternative variance estimation methods.

SI-1.1 Under-five mortality estimates by source, region and period

| Source | Region | Period | Taylor linearization estimate  est [95% CI] | Bootstrap estimate  est [95% CI] |
| --- | --- | --- | --- | --- |
| DHS | Conflict | 2014–2017 | 0.055 [0.047; 0.064] | 0.055 [0.048; 0.062] |
|  |  | 2018–2019 | 0.054 [0.043; 0.065] | 0.055 [0.046; 0.066] |
|  |  | 2020–2022 | 0.046 [0.036; 0.055] | 0.043 [0.034; 0.054] |
|  | Non-conflict | 2014–2017 | 0.058 [0.050; 0.065] | 0.053 [0.047; 0.059] |
|  |  | 2018–2019 | 0.045 [0.036; 0.054] | 0.038 [0.031; 0.046] |
|  |  | 2020–2022 | 0.044 [0.035; 0.053] | 0.037 [0.030; 0.045] |
| MPS | Conflict | 2014–2017 | 0.060 [0.048; 0.072] | 0.061 [0.049; 0.074] |
|  |  | 2018–2019 | 0.048 [0.038; 0.057] | 0.047 [0.035; 0.059] |
|  |  | 2020–2022 | 0.082 [0.066; 0.099] | 0.084 [0.070; 0.098] |
|  | Non-conflict | 2014–2017 | 0.057 [0.046; 0.068] | 0.056 [0.046; 0.067] |
|  |  | 2018–2019 | 0.045 [0.036; 0.054] | 0.043 [0.034; 0.053] |
|  |  | 2020–2022 | 0.058 [0.047; 0.070] | 0.059 [0.049; 0.070] |

SI-1.2: Under-five mortality estimates by source, region and period

| Source | Region | Period | Taylor linearization estimate  (95% CI) | Bootstrap estimate  (95% CI) |
| --- | --- | --- | --- | --- |
| DHS | Conflict | 2016–2019 | 0.054 [0.045; 0.063] | 0.055 [0.048; 0.062] |
|  |  | 2020–2022 | 0.046 [0.036; 0.055] | 0.043 [0.034; 0.054] |
|  | Non-conflict | 2016–2019 | 0.049 [0.043; 0.056] | 0.043 [0.037; 0.048] |
|  |  | 2020–2022 | 0.044 [0.035; 0.053] | 0.037 [0.030; 0.045] |
| MPS | Conflict | 2016–2019 | 0.055 [0.044; 0.066] | 0.056 [0.045; 0.066] |
|  |  | 2020–2022 | 0.082 [0.066; 0.099] | 0.084 [0.070; 0.098] |
|  | Non-conflict | 2016–2019 | 0.049 [0.039; 0.059] | 0.048 [0.040; 0.056] |
|  |  | 2020–2022 | 0.058 [0.047; 0.070] | 0.059 [0.049; 0.070] |

SI-1.3: Under-five mortality estimates by source, region and period

| Source | Region | Period | Taylor linearization estimate  (95% CI) | Bootstrap estimate  (95% CI) |
| --- | --- | --- | --- | --- |
| DHS | High conflict | 2014–2017 | 0.054 [0.043; 0.065] | 0.051 [0.041; 0.062] |
|  |  | 2018–2019 | 0.055 [0.044; 0.067] | 0.052 [0.038; 0.069] |
|  |  | 2020–2022 | 0.051 [0.041; 0.062] | 0.051 [0.035; 0.068] |
|  | Moderate conflict | 2014–2017 | 0.056 [0.048; 0.065] | 0.057 [0.048; 0.067] |
|  |  | 2018–2019 | 0.053 [0.042; 0.064] | 0.057 [0.043; 0.071] |
|  |  | 2020–2022 | 0.042 [0.034; 0.051] | 0.039 [0.028; 0.050] |
|  | Non conflict | 2014–2017 | 0.058 [0.050; 0.065] | 0.053 [0.047; 0.059] |
|  |  | 2018–2019 | 0.045 [0.036; 0.054] | 0.038 [0.031; 0.046] |
|  |  | 2020–2022 | 0.044 [0.035; 0.053] | 0.037 [0.030; 0.045] |
| MPS | High conflict | 2014–2017 | 0.075 [0.060; 0.090] | 0.077 [0.055; 0.099] |
|  |  | 2018–2019 | 0.053 [0.042; 0.063] | 0.052 [0.035; 0.072] |
|  |  | 2020–2022 | 0.111 [0.089; 0.133] | 0.112 [0.085; 0.139] |
|  | Moderate conflict | 2014–2017 | 0.049 [0.039; 0.059] | 0.049 [0.034; 0.064] |
|  |  | 2018–2019 | 0.045 [0.036; 0.054] | 0.043 [0.030; 0.059] |
|  |  | 2020–2022 | 0.065 [0.052; 0.078] | 0.067 [0.050; 0.083] |
|  | Non conflict | 2014–2017 | 0.057 [0.046; 0.068] | 0.056 [0.046; 0.067] |
|  |  | 2018–2019 | 0.045 [0.036; 0.054] | 0.043 [0.034; 0.053] |
|  |  | 2020–2022 | 0.058 [0.047; 0.070] | 0.059 [0.049; 0.070] |

**Note:** Conflict areas include East, North, Sahel, Centre-North, Centre-East, and Boucle du Mouhoun, classified as high-risk (Sahel, North, East) or moderate-risk (Boucle du Mouhoun, Centre-East, Centre-North) based on insurgent-related deaths (Figure 2).

SI-1.4: Under-five mortality estimates by source, region and period

| Source | Region | Period | Taylor linearization estimate  (95% CI) | Bootstrap estimate  (95% CI) |
| --- | --- | --- | --- | --- |
| DHS | High conflict | 2016–2019 | 0.054 [0.043; 0.065] | 0.051 [0.041; 0.061] |
|  |  | 2020–2022 | 0.051 [0.041; 0.062] | 0.051 [0.035; 0.068] |
|  | Moderate conflict | 2016–2019 | 0.054 [0.044; 0.064] | 0.057 [0.048; 0.066] |
|  |  | 2020–2022 | 0.042 [0.034; 0.051] | 0.039 [0.028; 0.050] |
|  | Non conflict | 2016–2019 | 0.049 [0.043; 0.056] | 0.043 [0.037; 0.048] |
|  |  | 2020–2022 | 0.044 [0.035; 0.053] | 0.037 [0.030; 0.045] |
| MPS | High conflict | 2016–2019 | 0.065 [0.052; 0.077] | 0.065 [0.051; 0.081] |
|  |  | 2020–2022 | 0.111 [0.089; 0.133] | 0.112 [0.085; 0.139] |
|  | Moderate conflict | 2016–2019 | 0.049 [0.039; 0.059] | 0.049 [0.037; 0.062] |
|  |  | 2020–2022 | 0.065 [0.052; 0.078] | 0.067 [0.050; 0.083] |
|  | Non conflict | 2016–2019 | 0.049 [0.039; 0.059] | 0.048 [0.040; 0.056] |
|  |  | 2020–2022 | 0.058 [0.047; 0.070] | 0.059 [0.049; 0.070] |

**Note:** Conflict areas include East, North, Sahel, Centre-North, Centre-East, and Boucle du Mouhoun, classified as high-risk (Sahel, North, East) or moderate-risk (Boucle du Mouhoun, Centre-East, Centre-North) based on insurgent-related deaths (Figure 2).

## **SI-2 Sample composition and survey performance of the MPS**

This Supporting Information provides detailed results to complement the main analyses. It documents the composition of the MPS samples by study arm, compares respondents’ characteristics with those of the census and DHS populations, and reports key survey performance indicators, including response rates, cooperation rates, and operational metrics. Additional stratified analyses by security context are also presented to assess representativeness and potential coverage differences.

***Table SI-2.1: Composition of the MPS sample, by study arm, and comparison with characteristics of the population enumerated in the 2019-2020 census***

|  | EHCVM : heads of household | | | RGPH | RDD : population 15-64 years olds | | | RGPH |
| --- | --- | --- | --- | --- | --- | --- | --- | --- |
| Respondents caracteristics | **Unweighted** | **Weighted** | |  | **Unweighted** | **Weighted** | |  |
|  | **%** | **%** | **95% CI** | **%** | **%** | **%** | **95% CI** | **%** |
| *Sex* |  | | | |  | | | |
| Men | 76.7 | 84.0 | [82.0-85.7] | 84.0 | 34.3 | 46.2 | [44.7-47.8] | 46.3 |
| Women | 23.3 | 16.0 | [14.3-18.0] | 16.0 | 65.7 | 53.8 | [52.2-55.3] | 53.7 |
| *Age group* |  | | | |  | | | |
| 15-29 yrs | 17.5 | 22.9 | [20.7-25.1] | 22.6 | 41.5 | 43.7 | [42.2-45.3] | 43.7 |
| 30-49 yrs | 52.0 | 49.3 | [46.8-51.9] | 49.1 | 46.8 | 42.2 | [40.7-43.8] | 42.2 |
| 50 yrs &+ | 30.6 | 27.8 | [25.6-30.2] | 28.3 | 11.7 | 14.1 | [13.0-15.2] | 14.1 |
| *Education* |  | | | |  | | | |
| None | 52.0 | 69.5 | [67.1-71.8] | 72,2 | 44.9 | 67.6 | [66.1-69.0] | 69,8 |
| Primary | 20.2 | 11.2 | [9.7-12.9] | 10 | 16.5 | 11.5 | [10.5-12.5] | 9,6 |
| Secondary | 21.4 | 13.7 | [12.1-15.6] | 13,5 | 29.5 | 15.7 | [14.6-16.8] | 15,93 |
| Superior | 6.4 | 5.6 | [4.5-6.9] | 4,2 | 9.0 | 5.2 | [4.6-5.9] | 3,6 |
| *Marital status* |  | | | |  | | | |
| Married | 83.6 | 83.3 | [81.3-85.1] | 84,2 | 75.4 | 77.5 | [76.2-78.8] | 72,5 |
| Widowed | 5.5 | 5.0 | [4.0-6.2] | 1,1 | 3.3 | 3.6 | [3.1-4.3] | 3,4 |
| Divorced/sep | 0.8 | 0.7 | [0.4-1.3] | 5,6 | 0.8 | 0.7 | [0.5-1.0] | 0,9 |
| Single | 10.1 | 11.0 | [9.5-12.7] | 9,1 | 20.4 | 18.2 | [17.0-19.4] | 23,2 |
| *Place of residence* |  | | | |  | | | |
| Ouaga | 11.1 | 13.0 | [11.4-14.8] | 14,4 | 15.8 | 11.5 | [10.6-12.4] | 14,5 |
| Bobo | 6.1 | 3.4 | [2.6-4.4] | 5 | 6.7 | 3.8 | [3.3-4.4] | 5,2 |
| Others town | 40.2 | 13.5 | [12.0-15.3] | 11,9 | 14.3 | 9.3 | [8.5-10.2] | 11,4 |
| Rural | 42.6 | 70.1 | [67.7-72.3] | 68,7 | 63.2 | 75.4 | [74.1-76.6] | 69 |
| *Region of residence* |  | | | |  | | | |
| Boucle du Mouhoun | 7.1 | 10.5 | [9.0-12.2] | 9,6 | 6.8 | 10.6 | [9.6-11.7] | 9,5 |
| Cascades | 7.4 | 4.3 | [3.4-5.5] | 3,9 | 4.1 | 4.1 | [3.5-4.8] | 4,1 |
| Centre | 15.7 | 16.8 | [14.9-18.8] | 18,4 | 24.3 | 13.4 | [12.5-14.5] | 18 |
| Centre-Est | 7.8 | 7.9 | [6.6-9.4] | 7,7 | 6.9 | 8.1 | [7.3-9.1] | 7,4 |
| Centre-Nord | 7.9 | 7.6 | [6.4-9.1] | 7 | 9.2 | 8.5 | [7.6-9.4] | 7,3 |
| Centre-Ouest | 6.8 | 7.4 | [6.1-8.9] | 7,8 | 7.6 | 8.3 | [7.5-9.2] | 8 |
| Centre-Sud | 5.6 | 4.5 | [3.6-5.7] | 4 | 4.3 | 4.6 | [3.9-5.3] | 3,9 |
| Est | 7.1 | 8.5 | [7.2-10.1] | 7,5 | 4.0 | 8.3 | [7.5-9.3] | 7,8 |
| Hauts-Bassins | 12.7 | 12.0 | [10.4-13.7] | 11,8 | 14.2 | 11.0 | [10.1-11.9] | 12 |
| Nord | 6.5 | 7.7 | [6.4-9.2] | 7,8 | 7.4 | 8.5 | [7.6-9.4] | 8,1 |
| Plateau-Central | 6.3 | 4.5 | [3.6-5.7] | 4,6 | 6.2 | 4.8 | [4.2-5.6] | 4,7 |
| Sahel | 2.9 | 3.8 | [2.9-4.9] | 5,3 | 1.7 | 5.4 | [4.7-6.2] | 4,6 |
| Sud-Ouest | 6.2 | 4.4 | [3.5-5.6] | 4,8 | 3.4 | 4.3 | [3.7-5.0] | 4,6 |
| *Household size* |  | | | |  | | | |
| 1-4 pers | 21.5 | 32.9 | [30.5-35.3] | 49.4 | 28.5 | 39.2 | [37.6-40.7] | 49.4 |
| 5-8 pers | 43.0 | 37.4 | [34.9-39.9] | 36.5 | 41.9 | 33.7 | [32.3-35.3] | 36.5 |
| 9 pers&+ | 35.4 | 29.7 | [27.4-32.1] | 14.1 | 29.6 | 27.1 | [25.7-28.6] | 14.1 |

***Table SI-2.2. Final disposition codes and phone call outcome rates by study arm***

|  | **EHCVM** | **RDD** |
| --- | --- | --- |
| **Total number of phone numbers used** | 8,809 | 43,841 |
| I=Complete interviews (1.1) | 5,087 | 11,176 |
| P=Partial interviews (1.2) | 100 | 4,063 |
| R=Refusal and break-off (2.1) | 457 | 2,181 |
| NC=Non-contact (2.2) | 3,039 | 15,035 |
| O=Other (2.0, 2.3) | 46 | 736 |
| UH=Unknown household (3.1) | 0 | 0 |
| UO=Unknown other (3.2–3.9) | 60 | 881 |
| **Response rates** |  | |
| Response rate 1: I/(I+P)+(R+NC+O)+(UH +OU) | 0.58 | 0.33 |
| Response rate 2: (I+P)/(I+P)+(R+NC+O)+(UH+OH) | 0.59 | 0.45 |
| **Cooperation rates** |  | |
| Cooperation rate 1[&3]: I/((I+P)+R+O) | 0.89 | 0.62 |
| Cooperation rate 2[&4]: (I+P)/((I+P)+R+O)) | 0.91 | 0.84 |
| **Refusal rates** |  | |
| Refusal rate 1: R/((I+P)+(R+NC+O)+UH+UO)) | 0.05 | 0.06 |
| Refusal rate 3: R/((I+P)+(R+NC+O)) | 0.06 | 0.07 |
| **Contact rates** |  | |
| Contact rate 1: (I+P)+R+O/(I+P)+R+O+NC+(UH+UO) | 0.65 | 0.53 |
| Contact rate 3: (I+P)+R+O/(I+P)+R+O+NC | 0.65 | 0.55 |
| **Survey length, costs, and productivity** |  | |
| Average survey length (mins) [min–max] | 23 [7–55] | 17 [7–45] |
| Average estimated cost per interview (USD) | 4 | 3 |
| Telephone numbers used to get an interview | 2 | 4 |

Note: EHCVM: Enquête harmonisée sur les conditions de vie des ménages; RDD: random digit dialing.

***Table SI-2.3: Comparison of women's characteristics by regional context of their place of residence in the MPS, DHS and the census***

|  | Conflict-affected provinces | | | | | | Non conflict-affected provinces | | | | | |
| --- | --- | --- | --- | --- | --- | --- | --- | --- | --- | --- | --- | --- |
|  | MPS | | | DHS | | Census | MPS | | | DHS | | Census |
|  | Unwgt | Weighted | | Weighted | |  | Unwgt | Weighted | | Weighted | |  |
|  | % | % | 95% CI | % | 95% CI | % | % | % | 95% CI | % | 95% CI | % |
| Age group |  |  |  |  |  |  |  |  |  |  |  |  |
| 15-29 yrs | 48.3 | 54.4 | [52.5-56.2] | 54.4 | [53.1-55.6] | 59.5 | 49.6 | 61.8 | [60.3-63.4] | 56.0 | [54.9-57.1] | 58.5 |
| 30-49 yrs | 51.7 | 45.6 | [43.8-47.5] | 45.6 | [44.4-46.9] | 40.5 | 50.4 | 38.2 | [36.6-39.7] | 44.0 | [42.9-45.1] | 41.5 |
|  |  |  |  |  |  |  |  |  |  |  |  |  |
| Education |  |  |  |  |  |  |  |  |  |  |  |  |
| None | 55.5 | 70.7 | [69.0-72.4] | 69.4 | [68.3-70.5] | 77.4 | 41.9 | 64.1 | [62.5-65.6] | 50.0 | [48.9-51.1] | 59.2 |
| Primary | 14.5 | 19.8 | [18.4-21.4] | 12.0 | [11.2-12.9] | 17.1 | 16.3 | 25.1 | [23.7-26.6] | 15.9 | [15.1-16.7] | 27.5 |
| Secondary & higher | 30.0 | 9.5 | [8.5-10.5] | 18.6 | [17.7-19.5] | 5.4 | 41.8 | 10.8 | [9.9-11.7] | 34.1 | [33.0-35.2] | 13.3 |
|  |  |  |  |  |  |  |  |  |  |  |  |  |
| Marital status |  |  |  |  |  |  |  |  |  |  |  |  |
| Married | 80.1 | 83.0 | [81.6-84.4] | 79.2 | [78.2-80.2] | 75.7 | 74.8 | 79.4 | [78.1-80.7] | 68.2 | [67.1-69.2] | 65.8 |
| Widowed | 2.6 | 3.4 | [2.8-4.2] | 1.3 | [1.0-1.6] | 1.8 | 2.6 | 3.3 | [2.8-4.0] | 1.8 | [1.5-2.0] | 2.1 |
| Divorced/sep | 0.9 | 0.9 | [0.6-1.4] | 1.1 | [0.9-1.3] | 0.8 | 1.0 | 0.8 | [0.6-1.2] | 2.3 | [2.0-2.7] | 1.0 |
| Single | 16.3 | 12.6 | [11.5-13.9] | 18.4 | [17.4-19.4] | 21.7 | 21.6 | 16.4 | [15.2-17.7] | 27.7 | [26.7-28.8] | 31.0 |
|  |  |  |  |  |  |  |  |  |  |  |  |  |
| Place of residence |  |  |  |  |  |  |  |  |  |  |  |  |
| Urban | 30.6 | 19.7 | [18.3-21.2] | 16.6 | [15.9-17.4] | 14.3 | 48.9 | 38.4 | [36.8-40.0] | 44.2 | [43.1-45.3] | 42.9 |
| Rural | 69.4 | 80.3 | [78.8-81.7] | 83.4 | [82.6-84.1] | 85.7 | 51.1 | 61.6 | [60.0-63.2] | 55.8 | [54.7-56.9] | 57.1 |
|  |  |  |  |  |  |  |  |  |  |  |  |  |
| Household size |  |  |  |  |  |  |  |  |  |  |  |  |
| 1-4 pers | 15.6 | 15.5 | [14.2-16.9] | 14.6 | [13.7-15.5] | 28.8 | 25.3 | 28.1 | [26.7-29.6] | 21.3 | [20.4-22.3] | 30.8 |
| 5-8 pers | 41.1 | 38.4 | [36.6-40.2] | 42.2 | [41.0-43.5] | 38.5 | 47.2 | 42.4 | [40.8-44.1] | 45.4 | [44.3-46.5] | 42.4 |
| 9 pers&+ | 43.3 | 46.2 | [44.3-48.0] | 43.2 | [41.9-44.5] | 32.7 | 27.4 | 29.4 | [27.9-31.0] | 33.3 | [32.3-34.3] | 26.8 |
|  |  |  |  |  |  |  |  |  |  |  |  |  |
| HH has electricity |  |  |  |  |  |  |  |  |  |  |  |  |
| Yes | 63.5 | 44.6 | [42.8-46.5] | 12.8 | [12.1-13.6] | 42.2 | 82.3 | 65.3 | [63.7-66.9] | 32.4 | [31.4-33.5] | 67.4 |
| No | 36.5 | 55.4 | [53.5-57.2] | 87.2 | [86.4-87.9] | 57.8 | 17.7 | 34.7 | [33.1-36.3] | 67.6 | [66.5-68.6] | 32.6 |
|  |  |  |  |  |  |  |  |  |  |  |  |  |
| Dwelling has durable roofing material |  |  |  |  |  |  |  |  |  |  |  |  |
| Yes | 84.3 | 77.0 | [75.3-78.5] | 82.7 | [81.6-83.6] | 72.5 | 95.0 | 90.9 | [89.9-91.9] | 90.2 | [89.6-90.9] | 88.4 |
| No | 15.7 | 23.0 | [21.5-24.7] | 17.3 | [16.4-18.4] | 27.5 | 5.0 | 9.1 | [8.1-10.1] | 9.8 | [9.1-10.4] | 11.6 |
|  |  |  |  |  |  |  |  |  |  |  |  |  |
| Household has access to an improved source of drinking water |  |  |  |  |  |  |  |  |  |  |  |  |
| Yes | 80.5 | 73.9 | [72.2-75.5] | 27.5 | [26.4-28.7] | 59.5 | 90.4 | 82.8 | [81.5-84.0] | 34.3 | [33.2-35.4] | 76.1 |
| No | 19.5 | 26.1 | [24.5-27.8] | 72.5 | [71.3-73.6] | 40.5 | 9.6 | 17.2 | [16.0-18.5] | 65.7 | [64.6-66.8] | 23.9 |

***Table SI-2.4: Comparison of women's characteristics according to the regional context of their place of residence using RaMMPS data, by study arm***

|  | Conflict | | | | | | Non conflict | | | | | |
| --- | --- | --- | --- | --- | --- | --- | --- | --- | --- | --- | --- | --- |
|  | RDD | | | EHCVM | | | RDD | | | EHCVM | | |
|  | Unw | Weighted | | Unw | Weighted | | Unw | Weighted | | Unw | Weighted | |
|  | % | % | 95% CI | % | % | 95% CI | % | % | 95% CI | % | % | 95% CI |
| Respondents age group |  |  |  |  |  |  |  |  |  |  |  |  |
| 15-29 yrs | 48.8 | 54.0 | [51.7-56.2] | 47.2 | 46.0 | [42.5-49.5] | 49.0 | 57.0 | [54.9-59.1] | 51.0 | 54.5 | [51.4-57.6] |
| 30-49 yrs | 51.2 | 46.0 | [43.8-48.3] | 52.8 | 54.0 | [50.5-57.5] | 51.0 | 43.0 | [40.9-45.1] | 49.0 | 45.5 | [42.4-48.6] |
|  |  |  |  |  |  |  |  |  |  |  |  |  |
| Education |  |  |  |  |  |  |  |  |  |  |  |  |
| None | 55.5 | 68.0 | [65.9-70.1] | 55.6 | 68.1 | [64.8-71.2] | 39.7 | 67.4 | [65.4-69.3] | 48.0 | 54.5 | [51.4-57.6] |
| Primary | 14.3 | 12.3 | [10.9-13.9] | 14.9 | 12.8 | [10.6-15.3] | 15.9 | 12.5 | [11.2-14.0] | 17.3 | 16.3 | [14.1-18.7] |
| Secondary | 27.1 | 18.2 | [16.5-20.0] | 27.5 | 18.8 | [16.3-21.7] | 34.3 | 16.3 | [14.8-17.8] | 31.1 | 25.5 | [22.9-28.2] |
| Higher | 3.1 | 1.5 | [1.0-2.1] | 2.0 | 0.4 | [0.1-0.9] | 10.1 | 3.8 | [3.2-4.6] | 3.6 | 3.8 | [2.7-5.2] |
|  |  |  |  |  |  |  |  |  |  |  |  |  |
| Marital status |  |  |  |  |  |  |  |  |  |  |  |  |
| Married | 81.1 | 83.2 | [81.5-84.9] | 78.1 | 83.7 | [81.0-86.1] | 74.6 | 79.4 | [77.7-81.1] | 75.4 | 79.6 | [77.0-81.9] |
| Widowed | 3.6 | 3.8 | [3.0-4.7] | 0.5 | 0.5 | [0.2-1.2] | 3.2 | 4.6 | [3.8-5.6] | 1.0 | 0.7 | [0.4-1.5] |
| Divorced/sep | 1.0 | 1.1 | [0.7-1.7] | 0.7 | 0.3 | [0.1-1.0] | 1.0 | 0.8 | [0.5-1.2] | 0.9 | 0.7 | [0.3-1.3] |
| Single | 14.3 | 11.9 | [10.5-13.5] | 20.6 | 15.5 | [13.1-18.2] | 21.3 | 15.2 | [13.7-16.8] | 22.6 | 19.0 | [16.7-21.5] |
|  |  |  |  |  |  |  |  |  |  |  |  |  |
| Place of residence |  |  |  |  |  |  |  |  |  |  |  |  |
| urban | 20.5 | 10.0 | [8.8-11.3] | 51.8 | 16.0 | [13.9-18.4] | 45.8 | 33.9 | [32.0-35.8] | 57.1 | 37.4 | [34.5-40.4] |
| rural | 79.5 | 90.0 | [88.7-91.2] | 48.2 | 84.0 | [81.6-86.1] | 54.2 | 66.1 | [64.2-68.0] | 42.9 | 62.6 | [59.6-65.5] |
|  |  |  |  |  |  |  |  |  |  |  |  |  |
| Household size |  |  |  |  |  |  |  |  |  |  |  |  |
| 1-4 members | 19.3 | 23.0 | [21.1-25.0] | 8.0 | 8.9 | [7.2-11.1] | 29.2 | 39.5 | [37.5-41.6] | 15.1 | 23.7 | [21.2-26.5] |
| 5-8 members | 41.1 | 39.5 | [37.3-41.7] | 41.0 | 36.2 | [32.9-39.6] | 47.2 | 38.9 | [36.8-41.0] | 47.3 | 43.2 | [40.1-46.3] |
| 9 members &+ | 39.6 | 37.6 | [35.4-39.8] | 51.1 | 54.9 | [51.4-58.3] | 23.6 | 21.6 | [19.9-23.4] | 37.7 | 33.1 | [30.2-36.0] |
|  |  |  |  |  |  |  |  |  |  |  |  |  |
| HH has electricity |  |  |  |  |  |  |  |  |  |  |  |  |
| Yes | 17.7 | 11.0 | [9.6-12.4] | 24.0 | 9.9 | [8.1-12.1] | 35.9 | 22.4 | [20.8-24.2] | 32.4 | 19.6 | [17.3-22.2] |
| No | 82.3 | 89.0 | [87.6-90.4] | 76.0 | 90.1 | [87.9-91.9] | 64.1 | 77.6 | [75.8-79.2] | 67.6 | 80.4 | [77.8-82.7] |
|  |  |  |  |  |  |  |  |  |  |  |  |  |
| Dwelling has durable roofing material |  |  |  |  |  |  |  |  |  |  |  |  |
| Yes | 85.8 | 77.7 | [75.7-79.5] | 81.3 | 70.7 | [67.4-73.8] | 95.0 | 87.0 | [85.4-88.4] | 94.9 | 90.6 | [88.5-92.2] |
| No | 14.2 | 22.3 | [20.5-24.3] | 18.7 | 29.3 | [26.2-32.6] | 5.0 | 13.0 | [11.6-14.6] | 5.1 | 9.4 | [7.8-11.5] |
|  |  |  |  |  |  |  |  |  |  |  |  |  |
| Household has access to an improved source of drinking water |  |  |  |  |  |  |  |  |  |  |  |  |
| Yes | 80.0 | 67.8 | [65.6-69.9] | 81.6 | 70.5 | [67.3-73.6] | 91.1 | 75.6 | [73.6-77.4] | 88.6 | 78.1 | [75.4-80.5] |
| No | 20.0 | 32.2 | [30.1-34.4] | 18.4 | 29.5 | [26.4-32.7] | 8.9 | 24.4 | [22.6-26.4] | 11.4 | 21.9 | [19.5-24.6] |

## SI-3: Sensitivity analyses: phone ownership, sample restrictions, conflict definitions, periods and weighting strategies

This section examines the sensitivity of under-five mortality estimates to our analytical choices, including differential phone ownership and reachability, restrictions to comparable households, alternative definitions of conflict exposure, period aggregation, and the use of different weighting and adjustment strategies.

## 1. Inverse probability weighting to adjust for differential phone reachability

To mitigate potential biases related to differential rates of successful phone recontact, we investigated the use of EHCVM surveys and sample harmonization techniques. Specifically, we implemented an alternative weighting scheme based on propensity score weighting to adjust for the probability of inclusion in the phone survey sample, and then compared the resulting estimates with those obtained using our initial weighting approach.

Our investigation used information from the original face-to-face EHCVM survey to better understand the characteristics of households reachable by phone and to address potential bias arising from differential phone reachability.

For this study, phone reachability refers to whether a household could be contacted and matched to a valid phone number in the survey data, not simply to phone ownership. The EHCVM data allowed us to distinguish between reachable and unreachable households.

We first used the EHCVM data to model reachability using a statistical model to estimate the probability of a household being reachable by phone based on observable characteristics. These included place of residence (urban/rural), region, household size, access to electricity, housing conditions (roofing), water source, and socio-demographic characteristics (see table SI-3.1 for variables used and statistics). This step produced a predicted probability of being recontacted by phone for each household.

We then applied this model to the MPS data, calculating the predicted probability of phone reachability for each respondent. We used the inverse of this probability as a weight (inverse probability weighting, IPW). Respondents with a low probability of being reachable (and therefore underrepresented in the sample) receive a higher weight, allowing them to better represent similar households in the general population.

To ensure comparability, analyses were restricted to the common support region, meaning that we only included observations whose predicted probabilities fell within the range observed among reachable households in the EHCVM. This avoids extrapolating beyond the support of the data.

For the DHS data, we retained the original sampling weights. These weights are provided with the survey and already account for the sampling design (e.g., unequal probabilities of selection across regions and households), ensuring that the DHS sample is representative of the national population.

***Table SI-3.1: characteristics of respondents by phone ownership***

| Variable | Unreachable  n (%) | Reachable | p |
| --- | --- | --- | --- |
| n | 1695 | 4880 |  |
| Age group |  |  | <0.001 |
| 15-29 yrs | 225 (13.3) | 480 (9.8) |  |
| 30-49 yrs | 816 (48.1) | 2539 (52.0) |  |
| 50 yrs &+ | 654 (38.6) | 1861 (38.1) |  |
| Education |  |  | <0.001 |
| None | 1242 (73.3) | 3214 (65.9) |  |
| Primary | 199 (11.7) | 751 (15.4) |  |
| Secondary&+ | 254 (15.0) | 915 (18.8) |  |
| Place of residence = rural (%) | 1030 (60.8) | 2464 (50.5) | <0.001 |
| HH size (%) |  |  | 0.005 |
| 1-4 pers | 580 (34.2) | 1486 (30.5) |  |
| 5-8 pers | 767 (45.3) | 2253 (46.2) |  |
| 9 pers&+ | 348 (20.5) | 1141 (23.4) |  |
| Electricity = No (%) | 1278 (75.4) | 3391 (69.5) | <0.001 |
| Roofing = No (%) | 356 (21.0) | 515 (10.6) | <0.001 |
| Water = No (%) | 315 (18.6) | 741 (15.2) | 0.001 |

*Note: this table is derived from original face-to-face EHCVM survey*

***Table SI-3.2. Under-five mortality (5q0) by period, region, data source and weihgting method***

| **Period** | **Region** | **Weighting** | **DHS** | **MPS** |
| --- | --- | --- | --- | --- |
| 2014–2017 | Conflict | PS | 0.055 (0.047–0.064) | 0.060 (0.048–0.072) |
| 2018–2019 | Conflict | PS | 0.054 (0.043–0.065) | 0.048 (0.038–0.057) |
| 2020–2022 | Conflict | PS | 0.046 (0.036–0.055) | 0.082 (0.066–0.099) |
| 2014–2017 | Non conflict | PS | 0.058 (0.050–0.065) | 0.057 (0.046–0.068) |
| 2018–2019 | Non conflict | PS | 0.045 (0.036–0.054) | 0.045 (0.036–0.054) |
| 2020–2022 | Non conflict | PS | 0.044 (0.035–0.053) | 0.058 (0.047–0.070) |
| 2014–2017 | Conflict | IPW | 0.056 (0.047–0.064) | 0.055 (0.044–0.066) |
| 2018–2019 | Conflict | IPW | 0.054 (0.043–0.065) | 0.046 (0.037–0.055) |
| 2020–2022 | Conflict | IPW | 0.046 (0.036–0.055) | 0.072 (0.058–0.086) |
| 2014–2017 | Non conflict | IPW | 0.058 (0.050–0.065) | 0.051 (0.041–0.061) |
| 2018–2019 | Non conflict | IPW | 0.045 (0.036–0.054) | 0.044 (0.035–0.052) |
| 2020–2022 | Non conflict | IPW | 0.044 (0.035–0.053) | 0.050 (0.040–0.059) |

*Note : PS : Post-stratification weighting; IPW: Inverse probability weighting*

## 2. Sensitivity to the definition of conflict-affected areas and time periods

***Figure SI-3.1: Sensitivity of under-five mortality estimates across data sources, conflict levels, and periods***

**Note:** Conflict areas include East, North, Sahel, Centre-North, Centre-East, and Boucle du Mouhoun, classified as high-risk (Sahel, North, East) or moderate-risk (Boucle du Mouhoun, Centre-East, Centre-North) based on insurgent-related deaths (Figure 2).

***Figure SI-3.2: Sensitivity of under-five mortality estimates across data sources, conflict levels, and periods***

**Note:** Conflict areas include East, North, Sahel, Centre-North, Centre-East, and Boucle du Mouhoun, classified as high-risk (Sahel, North, East) or moderate-risk (Boucle du Mouhoun, Centre-East, Centre-North) based on insurgent-related deaths (Figure 2).

## **SI-4** Child and Infant mortality trends by regional security context and survey sampling method

The following supplementary figures provide additional evidence on child and infant mortality trends, stratified by regional security context and survey sampling approach.

***Figure SI-4.1: trends in child mortality (_4_q_1_) based on regional security context, derived from RaMMPS survey and 2021 DHS***

*Note:RD: Relative Difference ((MPS-DHS)/DHS)*100*

***Figure SI-4.2: trends in child mortality (_4_q_1_) based on regional security context, and survey sampling method***

*Note:RD: Relative Difference ((MPS-DHS)/DHS)*100*

***Figure SI-4.3: trends in infant mortality (_1_q_0_) based on regional security context, derived from RaMMPS survey and 2021 DHS***

***Figure SI-4.4: trends in infant mortality (_1_q_0_) based on regional security context, and survey sampling method***

## SI-5: Coverage of conflict-affected provinces in the MPS: distribution of surveyed children and child deaths by inclusion in the DHS sampling frame

***Table SI-5: percentage of surveyed and deceased children in MPS sample by inclusion in the DHS sampling frame***

|  | Province | |
| --- | --- | --- |
|  | **Excluded**  **% (count)** | **Included**  **% (count)** |
| Surveyed | 1.8 (204) | 98.2 (11 018) |
| Proportion of deceased | 5.4 (11) | 5.2 (544) |
|  | **Age at death** | |
| Less than 12 month | 27.3 (3) | 62.3 (339) |
| 12 months or more | 72.7 (8) | 37.7 (205) |
|  | **Year of death** | |
| 2020-2022 | 81.8 (9) | 40.4 (220) |
| 2015-2019 | 18.2 (2) | 59.6 (324) |
| Persons-years | | |
| 2020-2022 | 1,025.9 | 3,175.5 |
